# Long-term functional outcome and quality of life 2.5 years after thrombolysis in acute ischemic stroke

**DOI:** 10.1186/s42466-023-00291-3

**Published:** 2023-11-09

**Authors:** Marie Schäbitz, Leona Möller, Anja Friedrich, Nele Klein, Alkisti Kitsiou, Isabell Greeve, Anja Gerstner, Leonard Wulff, Wolf-Rüdiger Schäbitz, Lars Timmermann, Andreas Rogalewski

**Affiliations:** 1 Department of Neurology, Evangelisches Klinikum Bethel, University Hospital OWL of the University Bielefeld, Campus Bielefeld-Bethel, Burgsteig 13, 33617 Bielefeld, Germany; 2 Department of Neurology, Philipps University Marburg, Marburg, Germany; 3 Department of Psychology, Bielefeld University, Bielefeld, Germany; 4 Department of Neurology, Sankt Elisabeth Hospital Gütersloh, Catholic Hospital Association of East Westfalia (KHO), Gütersloh, Germany

**Keywords:** Stroke, Thrombolysis, Long-term outcome, Quality of life, HRQoL, Modified Rankin Scale, EQ-5D-5L, EQ-VAS

## Abstract

**Background:**

Evaluation of outcome after stroke is largely based on assessment of gross function 3 months after stroke onset using scales such as mRS. Cognitive or social functions, level of symptom burden or emotional health are not usually assessed, nor are data available on long-term functional outcomes years after stroke.

**Methods:**

Analysis of 1141 patients with AIS treated with IVT from two major German university hospitals between 2017 and 2020. Patient characteristics and short-term outcome were analysed from patient records. Long-term outcome of 228 patients with prior written informed consent was assessed via telephone survey using mRS and PROMs (EQ-5D-5L, EQ-VAS) 2.5 years after stroke.

**Results:**

Predictors of excellent to good long-term outcome were younger age, event to door time ≤ 2 h, NIHSS ≤ 6 on admission and NIHSS ≤ 6 after IVT. Stroke recurrence was a negative predictor. Predictors of excellent quality of life at 2.5 years included age < 73 years, lower NIHSS after IVT, absence of hypertension. Quality of life was rated in all dimensions with a medium score of 1 and a medium EQ-VAS of 70, representing the good general health status of this stroke population.

**Conclusion:**

Main predictors of an excellent to good long-term outcome and excellent QoL 2.5 years after stroke are younger age, lower NIHSS, and event to door time ≤ 2 h. Research on long-term outcome after disease and treatment is of utmost importance, as it has the ability to reveal the patient true functional outcome and quality of life and to provide information on the status of independence and self-esteem.

## Introduction

Intravenous thrombolysis (IVT) is the basic treatment for acute ischemic stroke (AIS) patients applicable to approximately 25–30% of a patient population in large stroke centers [2]. Efficacy of IVT has been proven in numerous clinical trials by demonstrating improvement in functional outcome measures such as the modified Rankin Scale (mRS) 3 months after the stroke event (see meta-analysis by [5]). This type of outcome assessment largely relies on the physicians´ judgement on the patients´ outcome based on the 6-point mRS scale 3 months after the stroke event [26]. The patient’s cognitive and/or social functions, the amount of symptom burden (e.g., fatigue) or emotional health (e.g., depression) are poorly represented by the mRS. In addition, information on long-term functional outcome of AIS patients treated with IVT years after the event are relatively scarce [21, 24, 29].

Nowadays, outcome assessments after disease and treatment are transforming. Patient-reported outcome measures (PROMs) in neurological diseases came into focus and are a novel way to judge the patient outcome by self-rating, independent of physicians´ interpretation and evaluation. Interestingly, PROMs emerged as standard outcome measure in other neurological diseases such as myasthenia gravis, where they even defined the primary endpoint for evaluating the therapeutic efficacy of drugs in recent phase III trials [14, 15]. In AIS patients such an approach is not established, although recently assessment and reporting of stroke patients´ quality of life has begun [12, 17, 27]. Recently, PROMs were reported to correlate with clinician and self-reported mRS and reliably represented the outcome after a mild stroke or transient ischemic attack at 90 days [25].

In this analysis of an AIS patient population treated with IVT in two large German tertiary stroke centers, we assessed 2.5-year functional outcome by mRS as well as PROMs by using the EuroQol Group 5-Dimension (EQ-5D-5L) and the EuroQol-visual analogue scale (EQ-VAS) and identified predictors of excellent and of poor long-term outcome.

## Methods

### Patient characteristics

We conducted a retrospective analysis of patient records from two major German hospitals (tertiary referral centre, Evangelisches Klinikum Bethel, University Hospital OWL of the University Bielefeld, and Department of neurology, University Hospital of Marburg). A total of 1141 patients with acute ischemic stroke (AIS) were identified between January 1st, 2017 and December 31st, 2020. All patients who received thrombolysis were included, regardless of the affected vascular territory. Patients who underwent additional endovascular thrombectomy were not included.

### Procedure

Patient characteristics were comprehensively assessed including demographics, aetiology of stroke, and cardiovascular risk factors.

The patients’ initial blood pressure figures were recorded upon admission. Laboratory parameters were recorded on admission including blood glucose level, HbA1c, cholesterol level, LDL level, and CRP figures. Pre-existing statin therapy was recorded as well as pre-existing antiplatelet therapy or oral anticoagulation. Stroke severity was assessed using the modified Rankin Scale and NIHSS on admission and after thrombolysis.

The recorded complication rates addressed intracerebral haemorrhages (regardless of size and symptoms), symptomatic haemorrhages, epileptic seizures, recurrent strokes or transient ischemic attacks (TIA) within the first 72 h, infections requiring treatment within the first 72 h, and acute onset delirium. Clinically documented diagnoses in the patient records were taken into account.

Furthermore, a long-term catamnesis was prospectively conducted via telephone. Written informed consent was required for participation in the telephone survey. Patients who declined to participate or did not return a signed consent form were not included in the analysis.

The telephone interview was carried out by medical students (M.S., N.K.) who had been trained to conduct the interview using a standardised questionnaire. The telephone survey was conducted in a standardised manner by means of personal individual interviews with the patients or their relatives. The interviews started with the survey of the current mRS, the individual scales of the EQ-5D-5L by means of EQ-5D-5L Telephone interview version developed for the German language. Subsequently, questions with predefined answers were asked about the patient's living situation, prestroke care level and current care level, occupation, relapse event, renewed inpatient stay and current medication.

In the telephone interview, the mRS, the EQ-5D-5L questionnaire by the EuroQol Group as well as the EuroQol-visual analogue scale EQ-VAS were collected. The European Quality of Life in Five Dimensions (EQ-5D) is one of the most preferred generic instruments for assessing HRQoL in various conditions. The development and results of the EQ-5D-5L have been described elsewhere in detail [13]. An EQ-5D-5L telephone interview version has also been developed by EuroQoL for use when participants cannot be physically present during the interview, but have access to a telephone [9]. It could be shown that telephone interviews in general are a valid method for assessing quality of life and functional capacity, especially of older people [19]. The EQ-5D-5L questionnaire measures generic health-related quality of life, with the five dimensions (1) mobility, (2) self-care, (3) usual activities, (4) pain/discomfort, and (5) anxiety/depression. Each dimension is rated on a scale that describes the degree of problems in that area (i.e. no problems to walk, slight problems, moderate problems, severe problems, or unable to walk) with lower scores indicating a better HRQoL [4, 8, 13, 16]. The EQ summary index was conducted using the German population-based algorithm [13, 20]. Furthermore, ongoing antiplatelet therapy, anticoagulation therapy, statin therapy, antihypertensive therapy, and antidepressant therapy were recorded.

### Data analysis

Data analysis was carried out using the Statistical Package for the Social Sciences (SPSS) version 25 (IBM, 2018). Descriptive statistics were displayed as mean ± standard deviation for continuous data and frequencies with percentages for categorical variables. Normal distribution of residuals was assessed via Shapiro–Wilk test with *p* < 0.05 indicating non-normal distribution. Homoscedasticity was assessed visually via q–q-plots.

In all calculations, a *p* value of less than 0.05 in the two-sided test indicated statistical significance. Demographic characteristics were compared by using parametric t-tests or non-parametric Mann–Whitney U tests, depending on normal distribution. In order to identify potential predictors for a later binary logistic regression, preliminary analyses were carried out for the five group differences (1) favourable outcome (mRS 0 or 1 vs. mRS ≥ 2) assessed by telephone interview, (2) favourable outcome (mRS 0 or 1 vs. mRS ≥ 2) at discharge, (3) poor outcome (mRS 5 or 6 vs. mRS ≤ 4) at discharge, (4) favourable outcome (EQ index ≥ 0.970 vs. EQ index < 0.970) assessed by telephone interview, and (5) favourable outcome (EQ VAS ≥ 75 vs. EQ VAS < 75) assessed by telephone interview.

Parametric t-tests or non-parametric Mann–Whitney U-tests were used for ordinal and interval dependent variables (e.g. blood pressure, serum glucose level, HbA1c); differences in categorical variables (e.g. hypertension, diabetes mellitus and hypercholesterolaemia) were assessed using chi-square tests. In a second step, all statistically significant variables from the preliminary analyses were entered into a linear regression model and tested for multicollinearity. Relevant multicollinearity was assumed when the variance inflation factor (VIF) was greater than 2. Removing individual predictors with high VIF ensured a model with low multicollinearity. Finally, we computed four binary logistic regression models with the enter method [ordinal variables were compared with the simple (first) method] in order to identify relevant predictors of good long-term functional outcome, favourable outcome at discharge, poor outcome at discharge, and long-term quality of life.

In order to correct for alpha error accumulation in multiple testing, *p* values were adjusted separately for each set of analyses (favourable outcome, HRQoL, mortality, recanalization) using the Bonferroni method (p_adj_ = p_obs_*k; where p_adj_: adjusted *p*-value, p_obs_: observed *p* value, k = number of comparisons) [3].

## Results

### Demographic characteristics

In total, 1141 patients (46.2% Females) were included, regardless of the affected vascular territory. Mean age of all patients was 74.2 ± 13.4 years (Minimum: 17, Maximum: 100, Median: 77 years).

228 patients (42.1% Females) or their relatives gave written informed consent to participate in a telephone survey on long-term follow-up and were included in the long-term follow-up analysis. Mean age was 72.4 ± 12.2 years (Minimum: 29, Maximum: 97, Median: 73 years). The mean follow-up interval was 2.5 ± 1.0 years (median: 906 days).

**Fig. 1:**
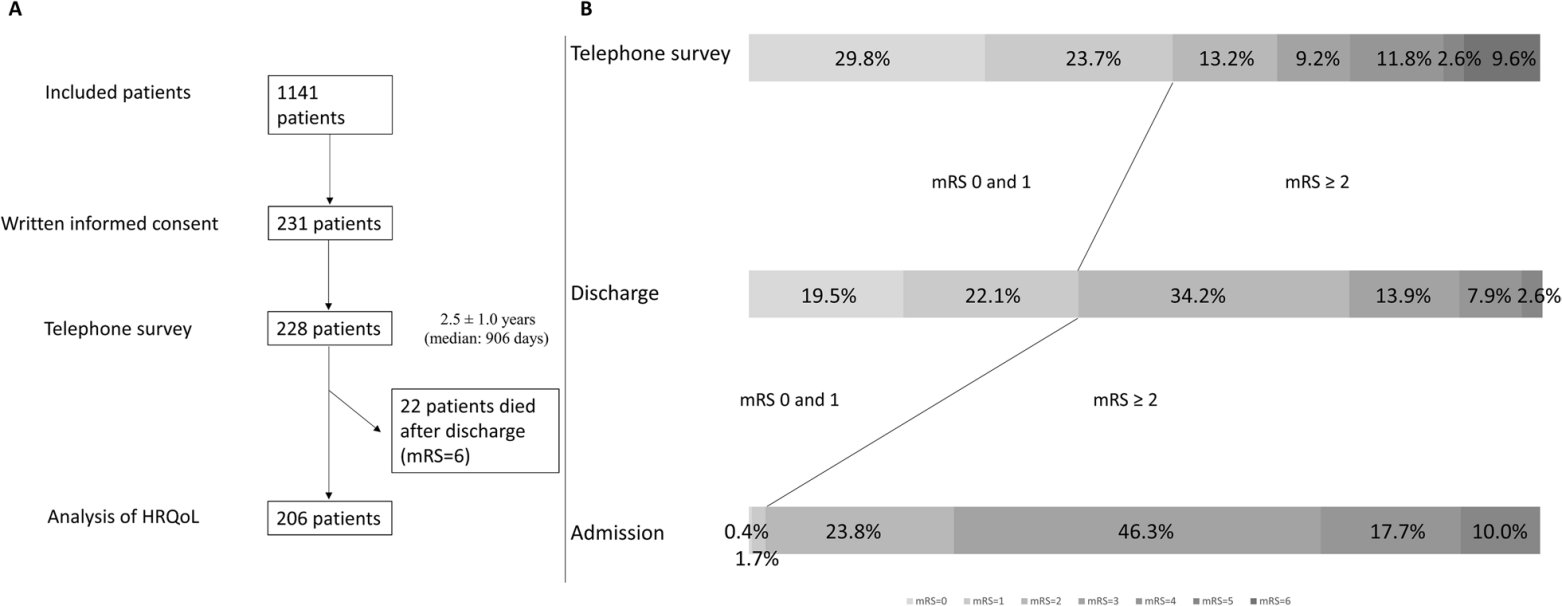
**A**: Presentation of the temporal order from inclusion of patients (N = 1141) via written informed consent (N = 231) to inclusion in the telephone survey (N = 228) is presented. It should be noted that the analysis of long-term follow-up by mRS included 228 patients inclusive of those who died in the meantime (mRS = 6), whereas the analysis of HRQoL included 206 patients without 22 patients with mRS = 6. **B**: Representation of the proportion of patients included at the telephone survey (N = 228) on admission, at discharge, and at timepoint of telephone survey according to the stroke severity measured by the modified Rankin Scale from 0 (light gray) to 5 (dark gray) on admission and at discharge as well as 0 to 6 at telephone survey

22 patients died (mRS = 6) after hospitalization (9.6%). These patients were included in the analyses of the mRS with an mRS score of 6 but were not included in the analyses on the current HRQoL, resulting in 206 patients analysed here. The temporal order of inclusion of patients via written informed consent and enrollment in the analyses is shown in Fig. 1A. Baseline demographic and clinical data as well as outcome parameters are summarised in Table 1.

**Table 1:** Long-term outcome parameters (N = 228)

*Sex*	
Female	96 (42.1%)
*Age*	
Mean 72.4 ± 12.2 [min 29; max 97], median 73 years	
*Interview partner*	
Patient	146 (64.0%)
Family member	81 (35.5%)
Caregiver	1 (0.4%)
Interval between stroke and telephone interview	
949.6 ± 358.9 days [min 303; max 2082 days]; median 906 days (= 2.5 years)	
*Long-term outcome of stroke* (N = 206)	
mRS at telephone interview	2.0 ± 1.9 (median 1)
Mobility EQ-5D-5L	2.0 ± 1.4 (median 1)
Self-care EQ-5D-5L	1.7 ± 1.3 (median 1)
Usual activities EQ-5D-5L	1.9 ± 1.4 (median 1)
Pain/Discomfort EQ-5D-5L	1.7 ± 1.1 (median 1)
Anxiety/Depression EQ-5D-5L	1.7 ± 1.0 (median 1)
EQ-5D VAS	64.5 ± 21.0 (median 70)
EQ-5D-Index	0.777 ± 0.287 (median 0.915)
*German level of care before stroke event* (N = 205)	
Level 0	180 (87.8%)
Level 1	3 (1.5%)
Level 2	13 (6.3%)
Level 3	6 (2.9%)
Level 4	3 (1.5%)
Level 5	0 (0%)
*German level of care at telephone interview* (N = 176)	
Level 0	109 (61.9%)
Level 1	12 (6.8%)
Level 2	19 (10.8%)
Level 3	19 (10.8%)
Level 4	13 (7.4%)
Level 5	4 (2.3%)
*Living situation* (N = 206)	
Independent	145 (70.4%)
Support from relatives or partner	29 (14.1%)
Care assistance at home	16 (7.8%)
Sheltered housing	5 (2.4%)
Care home	11 (5.3%)
*Professional activity* (N = 206)	
Unchanged after stroke	26 (12.6%)
Reduced due to stroke event	12 (5.8%)
No longer employed due to stroke event	8 (3.9%)
Retired (regardless of stroke event or retired before stroke event)	160 (77.7%)
*Recurrence of stroke* (N = 214)	
Recurrence	28 (13.1%)
No recurrence	186 (86.9%)
Unknown	14
*Therapy*	
Antiplatelet drugs	133/196 (67.9%)
Statin therapy	150/196 (76.5%)
Oral anticoagulation	57/196 (29.1%)
Phenprocoumon	4/57
Apixaban	35/57
Dabigatran	9/57
Edoxaban	3/57
Rivaroxaban	5/57
Unknown	1/57
Antihypertensive therapy	163/196 (83.2%)
Antidepressive therapy	31/196 (15.8%)
Anticonvulsive therapy	9/196 (4.6%)
*After discharge hospitalization for other reasons* (N = 199)	
Yes	61/199 (30.7%)
*Died after discharge*	
Yes	22/228 (9.6%)

The collective of patients participating in the telephone interview showed no significant difference in severity on admission compared to the overall collective (χ^2^ = 4.862, *p* = 0.433). At discharge, however, there was a higher severity and more number of patients who died in the overall collective compared to the telephone sample (χ^2^ = 26.111, *p* < 0.001). The distribution of severity on admission, at discharge and in the long-term outcome of the patients taking part in the telephone survey is shown in Fig. 1B.

## Predictors associated with favourable long-term outcome

Favourable long-term outcome was defined as a mRS score of 0 to 1 at the time of the telephone survey. 122 out of 228 patients had a mRS between 0 and 1 in the telephone survey (53.5%). Subsequently, comparisons were made between these 122 patients and those with mRS scores between 2 and 6 (n = 106).

### Identifying potential predictors for favourable long-term outcome

Patients with favourable long-term outcome were younger (67.9 ± 11.7 years vs. 77.5 ± 10.6, *p* < 0.001), less often females (36.1% vs. 49.1%, *p* = 0.048), had more often an event-to-door time ≤ 2 h (63.9% vs. 47.2%, *p* = 0.008), were less severely affected on admission (mRS: 2.8 ± 0.9 vs. 3.4 ± 0.9, *p* < 0.001; NIHSS: 4.9 ± 4.0 vs. 7.8 ± 7.1, *p* < 0.001) as well as after thrombolysis (NIHSS: 1.7 ± 2.6 vs. 5.1 ± 5.8, *p* < 0.001). Favourable long-term outcome was associated with less frequent hypertension (83.6% vs. 95.3%, *p* = 0.005), higher cholesterol levels on admission (203.0 ± 42.7 mg/dl vs. 188.7 ± 47.7 mg/dl, *p* = 0.033), less frequent former stroke (11.5% vs. 31.1%, *p* < 0.001), less frequent antiaggregant therapy on admission (25.6% vs. 48.1%, *p* < 0.001), less frequent occurrence of delirium during inpatient stay (2.5% vs. 8.5%, *p* = 0.042) and lower degree of German level of care prestroke (*p* < 0.001). Further results of analysis are displayed in Table 2.

**Table 2:** Predictors for a favorable outcome at telephone interview assessed by dichotomized mRS (mRS = 0 or 1 versus greater than or equal to 2) (N = 228)

	mRS = 0 or 1 (N = 122)	mRS ≥ 2 (N = 106)	Test statistics
Age	67.9 ± 11.7	77.5 ± 10.6	**U = 9,461.000, Z = 6.032, *p* < 0.001**^**c**^
Sex-Female	44/122 (36.1%)	52/106 (49.1%)	**3.927/0.048**^**a**^
*Aetiology of stroke*			
Large artery atherosclerosis stroke (LAAS)	14 (11.5%)	7 (6.7%)	4.531/0.997^a^
Cardioembolic stroke (CES)	28 (23.0%)	28 (26.7%)	
Cerebral small-vessel disease (SVD)	23 (18.9%)	21 (20.0%)	
Cryptogenic stroke	57 (46.7%)	49 (46.7%)	
Event to door time			
≤ 2 h	78/122 (63.9%)	50/106 (47.2%)	**6.474/0.008**^**a**^
Door to needle time	45.1 ± 25.2 min	46.6 ± 24.4 min	U = 6,847.000, Z = 0.767, *p* = 0.443^c^
	(Median 37)	(Median 40.5)	
*Severity indices on admission*			
Modified Rankin Scale on admission	2.8 ± 0.9	3.4 ± 0.9	**U = 8,269.000, Z = 3.865, *p* < 0.001**^**c**^
	Median: 3	Median: 3	
Modified Rankin Scale on admission	Score 0: 1 (0.8%)	Score 0: 0 (0%)	**19.425/< 0.001**^**a**^
	Score 1: 4 (3.3%)	Score 1: 0 (0%)	
	Score 2: 38 (31.1%)	Score 2: 17 (16.0%)	
	Score 3: 54 (44.3%)	Score 3: 51 (48.1%)	
	Score 4: 20 (16.4%)	Score 4: 20 (18.9%)	
	Score 5: 5 (4.1%)	Score 5: 18 (17.0%)	
NIHSS on admission	4.9 ± 4.0	7.8 ± 7.1	**U = 8,353.000, Z = 3.817, *p* < 0.001**^**c**^
	Median: 4	Median: 5.5	
NIHSS after 24 h	1.7 ± 2.6	5.1 ± 5.8	**U = 9,547.000, Z = 6.822, *p* < 0.001**^**c**^
	Median: 1	Median: 3	
*Risk factors*			
Hypertension	102/122 (83.6%)	101/106 (95.3%)	**7.921/0.005**^**a**^
Systolic blood pressure on admission	165.7 ± 25.7 mm Hg	168.2 ± 29.8 mm Hg	U = 6,586.500, Z = 0.495, *p* = 0.621^c^
Diastolic blood pressure on admission	90.8 ± 15.7 mm Hg	88.2 ± 15.1 mm Hg	U = 5,752.000, Z = 1.209, *p* = 0.227^c^
Diabetes mellitus	18/122 (14.8%)	22/106 (20.8%)	1.412/0.235^a^
Blood glucose level on admission	133.9 ± 55.6 mg/dl	135.0 ± 43.7 mg/dl	U = 6,957.000, Z = 1.102, *p* = 0.270^c^
HbA1c	41.7 ± 12.9 mmol/mol	42.8 ± 11.1 mmol/mol	U = 6,902.500, Z = 1.622, *p* = 0.105^c^
Cholesterol level on admission	203.0 ± 42.7 mg/dl	188.7 ± 47.7 mg/dl	**U = 5,207.500, Z = 2.135, *p* = 0.033**^**c**^
LDL level on admission	129.5 ± 37.9 mg/dl	118.5 ± 41.6 mg/dl	U = 5,273.000, Z = 1.888, *p* = 0.059^c^
Statine therapy on admission	36/121 (29.8%)	41/106 (38.7%)	2.009/0.156^a^
CRP level on admission	3.3 ± 5.8 mg/dl	10.8 ± 31.6 mg/dl	U = 7,230.500, Z = 1.657, *p* = 0.098^c^
Atrial fibrillation	19/122 (15.6%)	23/106 (21.7%)	1.416/0.234^a^
Former stroke	14/122 (11.5%)	33/106 (31.1%)	**13.392/< 0.001**^**a**^
Antiaggregant therapy on admission	31/121 (25.6%)	51/106 (48.1%)	**12.389/< 0.001**^**a**^
*Complications*			
Intracerebral bleeding	3/122 (2.5%)	7/106 (6.6.%)	3.529/0.317^a^
Seizure	2/122 (1.6%)	1/106 (0.9%)	0.212/0.646^a^
Recurrent stroke/TIA within 72 h	1/122 (0.8%)	1/106 (0.9%)	0.010/0.920^a^
Infection within 72 h	15/122 (12.3%)	21/106 (19.8%)	2.410/0.121^a^
Delirium	3/122 (2.5%)	9/106 (8.5%)	**4.138/0.042**^**a**^
*Pre-stroke living situation*			
German level of care (0–5)	0: 119 (97.5%)	0: 61 (73.5%)	**27.575/< 0.001**^**a**^
	1: 1 (0.8%)	1: 2 (2.4%)	
	2: 1 (0.8%)	2: 12 (14.5%)	
	3: 1 (0.8%)	3: 5 (6.0%)	
	4: 0 (0%)	4: 3 (3.6%)	
	5: 0 (0%)	5: 0 (0%)	

Parameters highlighted in bold indicate significant differences between the groups

^a^Chi square, ^b^parametric t-test, and ^c^Mann–Whitney U-Test used as appropriate

### Testing for multicollinearity and binary logistic regression for favourable long-term outcome

The parameter “prestroke German level of care” was not considered in the further analysis due to survey at late time point of telephone interview. The variance inflation factor was less than 1.446 for all variables. Predictors of good long-term outcome in binary logistic regression analysis comprised age (OR 1.081 [95% CI 1.060–1.102], *p* < 0.001), event to door time ≤ 2 h (OR 1.579 [95% CI 1.054–2.365], *p* = 0.027), NIHSS ≤ 6 on admission (OR 1.666 [95% CI 1.046–2.654], *p* = 0.032), and NIHSS ≤ 6 after thrombolysis (OR 5.717 [95% CI 2.869–11.393], *p* < 0.001). Former stroke was a negative predictor of good outcome (OR 0.552 [95% CI 0.330–0.922], *p* = 0.023) (Cox and Snell R^2^: 0.320; Nagelkerkes R^2^: 0.438; Hosmer–Lemeshow-Test: Chi-square 9.190, *p* = 0.327).

The previous analyses used the mRS in the long-term course as outcome parameter. However, this parameter gives little information about the quality of life, hence the following analyses were carried out with the EQ index of the EQ-5D-5L questionnaire by the EuroQol Group as well as the EuroQol-visual analogue scale EQ-VAS. An excellent quality of life score was defined as EQ index ≥ 0.970 or EQ-VAS ≥ 75. The results of the preliminary results can be seen in Table 3 (EQ index) and Table 4 (EQ-VAS).

**Table 3:** Predictors for a favorable outcome at telephone interview assessed by dichotomized EQ index of the EQ-5D-5L (EQ index ≥ 0.970 versus EQ index < 0.970) (N = 206)

	EQ index ≥ 0.970 (N = 83)	EQ index < 0.970 (N = 123)	Test statistics
Age	65.6 ± 11.4	74.7 ± 10.4	**U = 2,778.500, Z = 5.546, *p* < 0.001**^**c**^
Sex-Female	25/83 (30.1%)	56/123 (45.5%)	**4.931/0.026**^**a**^
*Aetiology of stroke*			
LAAS	9 (10.8%)	12 (9.8%)	2.482/0.779^a^
CES	22 (26.5%)	29 (23.8%)	
SVD	15 (18.1%)	22 (18.0%)	
Other	1 (1.2%)	1 (0.8%)	
Cryptogenic stroke	15 (18.1%)	33 (27.0%)	
ESUS	21 (25.3%)	25 (20.5%)	
Event to door time			
≤ 2 h	48/83 (57.8%)	70/123 (56.9%)	0.017/0.896^a^
Door to needle time	43.4 ± 26.1 min	44.5 ± 20.5 min	U = 4,574.500, Z = 1.263, *p* = 0.206^c^
	(Median 36)	(Median 38)	
*Severity indices on admission*			
Modified Rankin Scale on admission	2.9 ± 1.0	3.1 ± 0.9	U = 4,359.000, Z = 1.898, *p* = 0.058^c^
	Median: 3	Median: 3	
Modified Rankin Scale on admission	Score 0: 0 (0%)	Score 0: 1 (0.8%)	7.859/0.164^a^
	Score 1: 3 (3.6%)	Score 1: 1 (0.8%)	
	Score 2: 28 (33.7%)	Score 2: 25 (20.3%)	
	Score 3: 33 (39.8%)	Score 3: 63 (51.2%)	
	Score 4: 13 (15.7%)	Score 4: 22 (17.9%)	
	Score 5: 6 (7.2%)	Score 5: 11 (8.9%)	
NIHSS on admission	5.3 ± 4.3	6.0 ± 5.2	U = 4,678.000, Z = 1.022, *p* = 0.307^c^
	Median: 4	Median: 5	
NIHSS after 24 h	1.8 ± 2.7	3.5 ± 4.8	**U = 3,815.500, Z = 2.972, *p* = 0.003**^**c**^
	Median: 1	Median: 2	
*Risk factors*			
Hypertension	69/83 (83.1%)	112/123 (91.1%)	2.919/0.088^a^
Systolic blood pressure on admission	166.1 ± 25.1 mm Hg	167.1 ± 28.2 mm Hg	T = 0.259, *p* = 0.796^b^
Diastolic blood pressure on admission	91.8 ± 16.7 mm Hg	88.3 ± 14.8 mm Hg	U = 5,810.000, Z = 1.956, *p* = 0.050^c^
Diabetes mellitus	10/83 (12.0%)	26/123 (21.1%)	2.839/0.092^a^
Blood glucose level on admission	132.6 ± 51.0 mg/dl	136.5 ± 51.2 mg/dl	U = 4,687.500, Z = 0.854, *p* = 0.393^c^
HbA1c	40.9 ± 13.0 mmol/mol	42.6 ± 10.7 mmol/mol	**U = 3,819.000, Z = 2.574, *p* = 0.010**^**c**^
Cholesterol level on admission	204.8 ± 42.3 mg/dl	192.7 ± 46.1 mg/dl	T = 1.895, *p* = 0.060^b^
LDL level on admission	131.8 ± 35.3 mg/dl	120.6 ± 40.8 mg/dl	**U = 5,754.500, Z = 2.097, *p* = 0.036**^**c**^
Statine therapy on admission	21/82 (25.6%)	49/123 (39.8%)	**4.429/0.035**^**a**^
CRP level on admission	3.0 ± 5.1 mg/dl	9.1 ± 29.2 mg/dl	**U = 4,225.000, Z = 1.967, *p* = 0.049**^**c**^
Atrial fibrillation	11/83 (13.3%)	25/123 (20.3%)	1.719/0.190^a^
Former stroke	9/83 (10.8%)	29/123 (23.6%)	5.342/0.021^a^
Antiaggregant therapy on admission	21/83 (25.3%)	53/122 (43.4%)	**7.047/0.008**^**a**^
*Complications*			
Intracerebral bleeding	2/83 (2.4%)	6/123 (4.9.%)	1.155/0.764^a^
Seizure	1/83 (1.2%)	2/123 (1.6%)	0.061/0.805^a^
Recurrent stroke/TIA within 72 h	0/83 (0%)	2/123 (1.6%)	1.363/0.243^a^
Infection within 72 h	7/83 (8.4%)	22/123 (17.9%)	3.661/0.056^a^
Delirium	3/83 (3.6%)	7/123 (5.7%)	0.463/0.496^a^
*Pre-stroke living situation*			
German level of care (0–5)	0: 83 (100%)	0: 97 (79.5%)	**19.370/< 0.001**^**a**^
	1: 0 (0%)	1: 3 (2.5%)	
	2: 0 (0%)	2: 13 (10.7%)	
	3: 0 (0%)	3: 6 (4.9%)	
	4: 0 (0%)	4: 3 (2.5%)	
	5: 0 (0%)	5: 0 (0%)	
MRI parameters			

Parameters highlighted in bold indicate significant differences between the groups

^a^Chi square, ^b^parametric t-test, and ^c^Mann–Whitney U-Test used as appropriate

**Table 4:** Predictors for a favorable outcome at telephone interview assessed by dichotomized EQ VAS of the EQ-5D-5L (EQ VAS ≥ 75 versus EQ VAS < 75) (N = 206)

	EQ VAS ≥ 75 (N = 84)	EQ VAS < 75 (N = 122)	Test statistics
Age	68.0 ± 12.0	73.1 ± 11.1	**U = 3,875.000, Z = 2.972, *p* = 0.003**^**c**^
Sex-female	27/84 (32.1%)	54/122 (44.3%)	3.063/0.080^a^
*Aetiology of stroke*			
LAAS	9 (10.8%)	12 (9.8%)	0.817/0.976^a^
CES	21 (25.3%)	30 (24.6%)	
SVD	15 (18.1%)	22 (18.0%)	
Other	1 (1.2%)	1 (0.8%)	
Cryptogenic stroke	17 (20.5%)	31 (25.4%)	
ESUS	20 (24.1%)	26 (21.3%)	
Event to door time			
≤ 2 h	54/84 (64.3%)	64/122 (52.5%)	2.844/0.092^a^
Door to needle time	44.8 ± 26.0 min	43.6 ± 20.6 min	U = 5,000.000, Z = 0.295, *p* = 0.768^c^
	(Median 37)	(Median 38)	
*Severity indices on admission*			
Modified Rankin Scale on admission	2.8 ± 0.9	3.2 ± 0.9	**U = 4,170.000, Z = 2.424, *p* = 0.015**^**c**^
	Median: 3	Median: 3	
Modified Rankin Scale on admission	Score 0: 0 (0%)	Score 0: 1 (0.8%)	**11.479/0.043**^**a**^
	Score 1: 4 (4.8%)	Score 1: 0 (0%)	
	Score 2: 27 (32.1%)	Score 2: 26 (21.3%)	
	Score 3: 36 (42.9%)	Score 3: 60 (49.2%)	
	Score 4: 13 (15.5%)	Score 4: 22 (18.0%)	
	Score 5: 4 (4.8%)	Score 5: 13 (10.7%)	
NIHSS on admission	5.2 ± 4.4	6.1 ± 5.1	U = 4,411.500, Z = 1.704, *p* = 0.088^c^
	Median: 4	Median: 5	
NIHSS after 24 h	1.8 ± 2.8	3.6 ± 4.8	**U = 3,595.500, Z = 3.553, *p* < 0.001**^**c**^
	Median: 1	Median: 2	
*Risk factors*			
Hypertension	66/84 (78.6%)	115/122 (94.3%)	**11.486/< 0.001**^**a**^
Systolic blood pressure on admission	167.0 ± 28.3 mm Hg	166.3 ± 25.0 mm Hg	T = 0.173, *p* = 0.863^b^
Diastolic blood pressure on admission	89.3 ± 15.3 mm Hg	90.0 ± 15.9 mm Hg	U = 4,882.000, Z = 0.337, *p* = 0.736^c^
Diabetes mellitus	12/84 (14.3%)	24/122 (19.7%)	1.001/0.317^a^
Blood glucose level on admission	136.5 ± 53.8 mg/dl	133.9 ± 49.2 mg/dl	U = 5,215.000, Z = 0.365, *p* = 0.715^c^
HbA1c	41.8 ± 13.4 mmol/mol	42.0 ± 10.4 mmol/mol	U = 4,570.500, Z = 0.804, *p* = 0.421^c^
Cholesterol level on admission	201.2 ± 44.1 mg/dl	194.9 ± 45.3 mg/dl	U = 5,382.500, Z = 0.978, *p* = 0.328^c^
LDL level on admission	126.0 ± 38.3 mg/dl	124.5 ± 39.7 mg/dl	U = 5,057.500, Z = 0.291, *p* = 0.771^c^
Statine therapy on admission	23/83 (32.9%)	47/122 (38.5%)	2.569/0.109^a^
CRP level on admission	2.7 ± 5.0 mg/dl	9.3 ± 29.3 mg/dl	**U = 3,679.000, Z = 3.322, *p* < 0.001**^**c**^
Atrial fibrillation	12/84 (14.3%)	24/122 (19.7%)	1.001/0.317^a^
Former stroke	14/84 (16.7%)	24/122 (19.7%)	0.299/0.585^a^
Antiaggregant therapy on admission	19/82 (23.2%)	55/123 (44.7%)	9.900/0.002^a^
*Complications*			
Intracerebral bleeding	2/84 (2.4%)	6/122 (4.9.%)	1.203/0.752^a^
Seizure	1/84 (1.2%)	2/122 (1.6%)	0.070/0.792^a^
Recurrent stroke/TIA within 72 h	0/84 (0%)	2/122 (1.6%)	1.391/0.238^a^
Infection within 72 h	11/84 (13.1%)	18/122 (14.8%)	0.113/0.737^a^
Delirium	3/84 (3.6%)	7/122 (5.7%)	0.505/0.477^a^
*Pre-stroke living situation*			
German level of care (0–5)	0: 80 (95.2%)	0: 100 (82.6%)	8.249/0.083^a^
	1: 0 (0%)	1: 3 (2.5%)	
	2: 3 (3.6%)	2: 10 (8.3%)	
	3: 1 (1.2%)	3: 5 (4.1%)	
	4: 0 (0%)	4: 3 (2.5%)	
	5: 0 (0%)	5: 0 (0%)	

Parameters highlighted in bold indicate significant differences between the groups

^a^Chi square, ^b^parametric t-test, and ^c^Mann–Whitney U-Test used as appropriate

Predictor of excellent long-term quality of life measured by the EQ index in binary logistic regression analysis comprised age < 73 years (OR 2.629 [95% CI 1.311–5.273], *p* = 0.006) (Cox and Snell R^2^: 0.228; Nagelkerkes R^2^: 0.307; Hosmer–Lemeshow-Test: Chi-square 12.557, *p* = 0.128).

Predictors of excellent long-term quality of life measured by the EQ-VAS in binary logistic regression analysis comprised lower NIHSS after thrombolysis (OR 0.896 [95% CI 0.804–0.998], *p* = 0.046) and absence of hypertension (OR 2.836 [95% CI 1.063–7.562], *p* = 0.037) (Cox and Snell R^2^: 0.144; Nagelkerkes R^2^: 0.195; Hosmer–Lemeshow-Test: Chi-square 14.554, *p* = 0.068).

## Predictors for favourable and poor outcome at discharge

In further analyses, all 1141 patients in the total cohort were included and possible predictors of good outcome (mRS 0 or 1) and poor outcome (mRS 5 or 6) at discharge were analysed. The results of the results can be seen in Table 5 (mRS 0 or 1 vs. mRS 2–6) and Table 6 (mRS 5 or 6 vs. mRS 0–4). After testing for multicollinearity, binary logistic regression was performed for both favourable as well as poor outcome at discharge.

**Table 5:** Predictors for a favorable outcome at discharge assessed by dichotomized mRS (mRS = 0 or 1 versus greater than or equal to 2) (N = 1141)

	mRS = 0 or 1 (N = 406)	mRS ≥ 2 (N = 735)	Test statistics
Age	68.5 ± 14.0	77.3 ± 11.9	**U = 205,118.500, Z = 10.496, *p* < 0.001**^**c**^
Sex-female	167/406 (41.1%)	360/735 (49.0%)	**6.479/0.011**^**a**^
*Aetiology of stroke*			
LAAS	61 (15.1%)	105 (14.4%)	**16.258/0.006**^**a**^
CES	88 (21.7%)	226 (31.0%)	
SVD	92 (22.7%)	146 (20.0%)	
Other	3 (0.7%)	1 (0.1%)	
Cryptogenic stroke	94 (23.2%)	127 (17.4%)	
ESUS	67 (16.5%)	124 (17.0%)	
Event to door time			
≤ 2 h	248/406 (61.1%)	379/735 (51.6%)	**9.573/0.002**^**a**^
Door to needle time	46.6 ± 31.2 min	47.5 ± 25.6 min	**U = 158,605.000, Z = 1.989, *p* = 0.047**^**c**^
	(Median 38)	(Median 42)	
*Severity indices on admission*			
Modified Rankin Scale on admission	2.6 ± 0.9	3.5 ± 1.0	**U = 218,918.500, Z = 13.849, *p* < 0.001**^**c**^
	Median: 3	Median: 3	
Modified Rankin Scale on admission	Score 0: 4 (1.0%)	Score 0: 0 (0%)	**199.712/< 0.001**^**a**^
	Score 1: 17 (4.2%)	Score 1: 1 (0.1%)	
	Score 2: 159 (39.3%)	Score 2: 102 (13.9%)	
	Score 3: 178 (44.0%)	Score 3: 298 (40.5%)	
	Score 4: 34 (8.4%)	Score 4: 196 (26.7%)	
	Score 5: 13 (3.2%)	Score 5: 138 (18.8%)	
NIHSS on admission	4.6 ± 3.8	8.4 ± 6.1	**U = 215,598.000, Z = 12.602, *p* < 0.001**^**c**^
	Median: 4	Median: 6	
NIHSS after 24 h	1.3 ± 2.0	6.5 ± 6.3	**U = 237,571.000, Z = 19.497, *p* < 0.001**^**c**^
	Median: 1	Median: 4	
*Risk factors*			
Hypertension	344/406 (84.7%)	689/735 (93.7%)	**24.789/< 0.001**^**a**^
Systolic blood pressure on admission	162.8 ± 25.2 mm Hg	165.2 ± 28.8 mm Hg	U = 148,646.000, Z = 1.318, *p* = 0.187^c^
Diastolic blood pressure on admission	89.9 ± 14.6 mm Hg	88.7 ± 16.8 mm Hg	U = 133,361.500, Z = 1.663, *p* = 0.096^c^
Diabetes mellitus	75/406 (18.5%)	198/735 (26.9%)	**10.298/0.001**^**a**^
Blood glucose level on admission	130.8 ± 43.1 mg/dl	141.9 ± 56.6 mg/dl	**U = 167,538.000, Z = 3.482, *p* < 0.001**^**c**^
HbA1c	41.2 ± 9.6 mmol/mol	43.7 ± 13.6 mmol/mol	**U = 157,659.000, Z = 2.685, *p* = 0.007**^**c**^
Cholesterol level on admission	202.0 ± 49.2 mg/dl	195.6 ± 50.8 mg/dl	**U = 132,558.000, Z = 2.206, *p* = 0.027**^**c**^
LDL level on admission	124.3 ± 44.8 mg/dl	124.3 ± 42.5 mg/dl	U = 134,583.000, Z = 1.536, *p* = 0.125^c^
Statine therapy on admission	119/404 (29.5%)	223/730 (30.5%)	0.147/0.701^a^
CRP level on admission	5.1 ± 12.7 mg/dl	11.1 ± 27.1 mg/dl	**U = 175,232.500, Z = 4.929, *p* < 0.001**^**c**^
Atrial fibrillation	56/406 (13.8%)	203/735 (27.6%)	**28.492/< 0.001**^**a**^
Former stroke	83/406 (20.4%)	192/735 (26.1%)	**4.611/0.032**^**a**^
Antiaggregant therapy on admission	142/402 (35.3%)	314/730 (43.0%)	**6.373/0.012**^**a**^
*Complications*			
Intracerebral bleeding	12/406 (3.0%)	70/734 (9.5.%)	**17.362/0.002**^**a**^
Seizure	4/406 (1.0%)	19/735 (2.6%)	3.389/0.066^a^
Recurrent stroke/TIA within 72 h	0/406 (0%)	3/735 (0.4%)	0.010/0.920^a^
Infection within 72 h	37/406 (9.1%)	233/735 (31.7%)	**73.867/< 0.001**^**a**^
Delirium	5/406 (1.2%)	68/735 (9.3%)	**28.091/< 0.001**^**a**^
*Pre-stroke living situation*			
German level of care (0–5)	0: 197 (95.2%)	0: 209 (76.0%)	**33.040/< 0.001**^**a**^
	1: 1 (0.5%)	1: 6 (2.2%)	
	2: 4 (1.9%)	2: 35 (12.7%)	
	3: 4 (1.9%)	3: 18 (6.5%)	
	4: 1 (0.5%)	4: 7 (2.5%)	
	5: 0 (0%)	5: 0 (0%)	

Parameters highlighted in bold indicate significant differences between the groups

^a^Chi square, ^b^parametric t-test, and ^c^Mann–Whitney U-Test used as appropriate

**Table 6:** Predictors for a poor outcome at discharge assessed by dichotomized mRS (mRS = 5 or 6 versus lower than or equal to 4) (N = 1141)

	mRS = 0 to 4 (N = 1025)	mRS ≥ 5 (N = 116)	Test statistics
Age	73.2 ± 13.4	83.1 ± 8.4	**U = 86,889.000, Z = 8.160, *p* < 0.001**^**c**^
Sex-female	460/1025 (44.9%)	67/116 (57.8%)	**6.956/0.008**^**a**^
*Aetiology of stroke*			
LAAS	145 (14.2%)	21 (18.9%)	31**.765/< 0.001**^**a**^
CES	262 (25.6%)	52 (46.8%)	
SVD	229 (22.4%)	9 (8.1%)	
Other	4 (0.4%)	0 (0%)	
Cryptogenic stroke	204 (19.9%)	17 (15.3%)	
ESUS	179 (17.5%)	12 (10.8%)	
Event to door time			
≤ 2 h	574/1025 (56.0%)	53/116 (45.7%)	**4.475/0.034**^**a**^
Door to needle time	46.7 ± 27.1 min	51.4 ± 32.4 min	U = 63,468.500, Z = 1.409, *p* = 0.159^c^
	(Median 40)	(Median 42)	
*Severity indices on admission*			
Modified Rankin Scale on admission	3.1 ± 0.9	4.3 ± 0.9	**U = 96,140.000, Z = 11.496, *p* < 0.001**^**c**^
	Median: 3	Median: 5	
Modified Rankin Scale on admission	Score 0: 4 (0.4%)	Score 0: 0 (0%)	**190.277/< 0.001**^**a**^
	Score 1: 18 (1.8%)	Score 1: 0 (0%)	
	Score 2: 257 (25.1%)	Score 2: 4 (3.4%)	
	Score 3: 456 (44.5%)	Score 3: 20 (17.2%)	
	Score 4: 198 (19.3%)	Score 4: 32 (27.6%)	
	Score 5: 91 (8.9%)	Score 5: 60 (51.7%)	
NIHSS on admission	6.3 ± 4.8	13.9 ± 7.6	**U = 97,796.000, Z = 11.476, *p* < 0.001**^**c**^
	Median: 5	Median: 14	
NIHSS after 24 h	3.6 ± 4.2	16.3 ± 8.1	**U = 83,353.000, Z = 13.521, *p* < 0.001**^**c**^
	Median: 2	Median: 16	
*Risk factors*			
Hypertension	921/1025 (89.9%)	112/116 (96.6%)	**5.456/0.020**^**a**^
Systolic blood pressure on admission	164.4 ± 27.2 mm Hg	163.8 ± 30.5 mm Hg	U = 54,982.000, Z = 0.299, *p* = 0.765^c^
Diastolic blood pressure on admission	89.2 ± 15.7 mm Hg	88.4 ± 19.5 mm Hg	U = 52,006.000, Z = 1.224, *p* = 0.221^c^
Diabetes mellitus	245/1025 (23.9%)	28/116 (24.1%)	0.003/0.955^a^
Blood glucose level on admission	135.8 ± 47.4 mg/dl	156.4 ± 82.6 mg/dl	**U = 67,353.500, Z = 2.369, *p* = 0.018**^**c**^
HbA1c	42.6 ± 12.2 mmol/mol	44.2 ± 14.6 mmol/mol	U = 54,029.000, Z = 0.160, *p* = 0.873^c^
Cholesterol level on admission	198.5 ± 49.7 mg/dl	192.6 ± 55.4 mg/dl	U = 50,652.500, Z = 1.386, *p* = 0.166^c^
LDL level on admission	126.2 ± 43.2 mg/dl	125.0 ± 44.9 mg/dl	U = 52,609.000, Z = 0.400, *p* = 0.689^c^
Statine therapy on admission	313/1020 (30.7%)	29/114 (25.4%)	1.341/0.247^a^
CRP level on admission	7.6 ± 19.3 mg/dl	21.3 ± 42.9 mg/dl	**U = 77,073.500, Z = 5.262, *p* < 0.001**^**c**^
Atrial fibrillation	210/1025 (20.5%)	49/116 (42.2%)	**28.103/< 0.001**^**a**^
Former stroke	242/1025 (23.6%)	33/116 (28.4%)	1.334/0.248^a^
Antiaggregant therapy on admission	406/1017 (39.9%)	50/115 (43.5%)	0.543/0.461^a^
*Complications*			
Intracerebral bleeding	59/1025 (5.8%)	23/115 (20.0.%)	**36.484/< 0.001**^**a**^
Seizure	14/1025 (1.4%)	9/116 (7.8%)	**21.561/< 0.001**^**a**^
Recurrent stroke/TIA within 72 h	2/1025 (0.2%)	1/116 (0.9%)	1.768/0.184^a^
Infection within 72 h	202/1025 (19.7%)	68/116 (58.6%)	**87.354/< 0.001**^**a**^
Delirium	63/1025 (6.1%)	10/116 (8.6%)	1.065/0.302^a^
*Pre-stroke living situation*			
German level of care (0–5)	0: 400 (85.1%)	0: 6 (50.0%)	**17.571/0.001**^**a**^
	1: 7 (1.5%)	1: 0 (0%)	
	2: 37 (7.9%)	2: 2 (16.7%)	
	3: 19 (4.0%)	3: 3 (25.0%)	
	4: 7 (1.5%)	4: 1 (8.3%)	
	5: 0 (0%)	5: 0 (0%)	

Parameters highlighted in bold indicate significant differences between the groups

^a^Chi square, ^b^parametric t-test, and ^c^Mann–Whitney U-Test used as appropriate

Predictors of favourable outcome at discharge in binary logistic regression analysis comprised age (OR 1.033 [95% CI 1.020–1.046], *p* < 0.001), mRS 0 or 1 on admission (OR 18.048 [95% CI 2.356–138.254], *p* = 0.005), NIHSS ≤ 6 after thrombolysis (OR 9.682 [95% CI 5.315–17.636], *p* < 0.001). Infection within 72 h (OR 0.533 [95% CI 0.344–0.826], *p* = 0.005) as well as delirium (OR 0.256 [95% CI 0.098–0.670], *p* = 0.006) were negative predictors of favourable outcome at discharge (Cox and Snell R^2^: 0.245; Nagelkerkes R^2^: 0.336; Hosmer–Lemeshow-Test: Chi-square 3.018, *p* = 0.933).

Predictors of poor outcome at discharge in binary logistic regression analysis comprised higher age (OR 1.075 [95% CI 1.037–1.114], *p* < 0.001), NIHSS > 9 after thrombolysis (OR 21.928 [95% CI 11.888–40.447], *p* < 0.001), elevated serum glucose level on admission (OR 1.005 [95% CI 1.001–1.009], *p* = 0.024), elevated CRP level on admission (OR 1.010 [95% CI 1.002–1.019], *p* = 0.019) and infection within 72 h (OR 2.347 [95% CI 1.332–4.135], *p* = 0.003) (Cox & Snell R^2^: 0.218; Nagelkerkes R^2^: 0.506; Hosmer–Lemeshow-Test: Chi-square 7.276, *p* = 0.507).

## Discussion

### Longterm outcome and predictors

Our data represent the results of the largest cohort of patients treated with thrombolysis and followed for long-term functional outcome assessment by mRS and quality of life using PROMs reported so far. Predictors of excellent to good long-term outcome in this study are lower age, an event to door time ≤ 2 h, a NIHSS ≤ 6 on admission and a NIHSS ≤ 6 after thrombolysis, whereas stroke recurrence was a negative predictor. Predictors of excellent quality of life after 2.5 years included age < 73 years, lower NIHSS after IVT and absence of hypertension.

Very recently, comparable predictors of a good quality of Life (QoL) were reported with age < 75 years, NIHSS score ≤ 4, and modified Rankin Scale score ≤ 2 at 6 and 12 months after stroke [7]. Surprisingly, revascularization therapy including IVT had no significant effect on the QoL scores in this study. A small study with 88 patients also reported no major differences in health related QoL between IVT and untreated patients 1 year after stroke [6]. These findings are largely supported by a recent study demonstrating that initial functional deficits, age and recurrent strokes predict health related QoL, whereas acute therapies including IVT beyond their immediate effect were not clearly associated with PROMs [17]. Interesting in this study is the finding that the subjective health related QoL steadily increased for all patients and recovered to high levels at 12 months despite a high proportion of persisting disability in up to 29% of the patients. In particular severely affected patients needed longer periods of time and improved most between 3 and 12 months [17]. Longer intervals for follow up of at least 2-years are warranted to assure a stable and representative status for final functional outcome evaluation.

The long-term 2.5-year outcome as reported in our study was analyzed by PROMs, assessed by telephone interview based on the established EQ-5D-5L and the EQ-VAS scores for measuring quality of life after stroke [12, 27]. The EQ-5D-5L is based on the five dimensions mobility, self-care, usual activities, pain/discomfort and anxiety/depression. Patients were asked to rank their current health condition into five levels defined as no problems, slight problems, moderate problems, severe problems, or unable to take part in this dimension. Patients in our study rated their quality of life in all dimensions with a medium score of 1, which means no problems concerning mobility, self-care and usual activities and no pain/discomfort or anxiety/depression (see Table 1). This is indicative of the general good QoL and supports the impression of the overall good outcome of this stroke population. The EQ-VAS is a scale from 1 to 100, with 100 being the best health status possible and 1 being the worst, irrespective of parameters like age, comorbidities or current disease. Patients in the present study showed a medium EQ-VAS of 70 representing a good general health status further supporting the perception of an overall good long-term outcome of this stroke population (see Table 1).

Outcome assessment also included the pre-stroke and post-stroke German levels of care (level 0 no support, fully independence to level 5 full support, complete dependence). Most patients in our study (87.8%) had a pre-stroke level of 0. 61.9% of patients still lived at level 0 and 6.8% at level 1 indicative of the overall good outcome and quality of life 2.5 years after stroke and thrombolysis (Table 1). This finding was supported by the current living situation: 70.1% of patients of this stroke population is currently living independently at home or gets support from relatives or partner (14.1%, see Table 1). The results of our study underline the importance of regaining independence and self-esteem for a good or excellent recovery and a higher quality of life in the long-term.

### Predictors for a good and poor outcome at discharge

In our study, positive predictors of good outcome at discharge, defined as mRS 0–1 were lower age, mRS 0 or 1 on admission and NIHSS ≤ 6 24 h after thrombolysis—which is in line with previous reports [1, 28]. Consistently, high age and NIHSS > 9 24 h after thrombolysis, predicted a poor outcome at discharge defined as mRS 5 or 6. Patients with infections such as pneumonia after stroke are more likely to get further complications during the acute phase and are at higher risk for worse outcome and mortality [22, 30]. This is further supported by our study which confirmed infections within 72 h after stroke onset as a negative predictor for a good outcome at discharge, as well as high CRP levels at admission to be predictive for a poor outcome at discharge.

Importantly, our study identified post-stroke delirium for the first time in patients treated with thrombolysis as a negative predictor for a good outcome. Post-stroke delirium is not widely recognized as negative outcome determining factor, and supporting data are scarce. In a recent prospective study on 227 patients treated on a Stroke Unit, delirium occurred in 71 patients (31.3%) and was associated with increased risk of death and functional dependence at 30 and 90 days and higher 90-day mortality [10]. Retrospective data of 564 patients of a broad stroke population including TIA reported the occurrence of delirium in 23.4% of patients within 7 days after stroke onset to be associated with a higher 5-year mortality [18].

### Study limitations

A study immanent limitation represents the selection bias regarding good outcome. The reason for this is twofold: Due to strict protection of data privacy patients could only be contacted after receiving and signing declaration of consent—producing a bias towards a population of responsive and health-oriented patients. On the other hand, QoL was measured by a self-assessment via telephone interview requiring the patient or its relative to be able to talk on the phone. Patients with poor health conditions living in a nursing home or getting professional nursing care at home are presumably underrepresented. In addition, a recurrent stroke was a relatively rare event in patients participating in the telephone interview (13.1%). This may be explained to some extent by the poor health condition in patients with one or more recurrent strokes resulting in the impossibility to participate. Design and conductance of the present study type is prone for a selection bias towards healthier patients which is immanent for this type of studies and independent of the presented one.

It could be shown that telephone interviews are a valid method for assessing quality of life and functional capacity, especially of older people [19]. In addition, reduced participation has been observed in all epidemiological study designs, both in the form of non-response and refusal in recent years [23]. Glass et al. identified factors affecting willingness to participate in health research telephone surveys [11]. Participants in previous studies, older people and women were shown to be the groups most likely to participate. Younger men preferred online surveys, older people preferred a written questionnaire, and few participants of all ages and gender groups preferred a telephone questionnaire.

## Conclusion

In conclusion, our data represent the results of the largest cohort of patients treated with thrombolysis and followed for long-term functional outcome assessed by mRS and quality of life assessed by PROMs reported to date. The main predictors of excellent to good long-term outcome and excellent QoL 2.5 years after stroke are younger age, lower NIHSS and event to door time ≤ 2 h. Overall QoL was surprisingly good, indicating a good general health status of this stroke population. A major limitation of the study is the fact that it was not possible to contact patients without prior written consent due to data protection regulations and the requirements of the ethics vote. As a result, only about 20% of the total cohort participated in the telephone interview, which may lead to selection bias and reduce statistical power. However, research on long-term outcomes after disease and treatment is of paramount importance as it has the ability to reveal the true functional outcome and quality of life of the patient and provide information on the status of independence and self-esteem.

## Data Availability

The data that support the findings of this study are available from the corresponding author upon reasonable request.

## Abbreviations

IVT: Intravenous thrombolysis
AIS: Acute ischemic stroke
mRS: Modified Rankin Scale
PROMs: Patient-reported outcome measures
HRQoL: Health-related quality of life
EQ-5D: EuroQol Group 5-Dimension
EQ-VAS: EuroQol-visual analogue scale
QoL: Quality of Life

## Code availability

Statistical analysis of the data was carried out using the Statistical Package for the Social Sciences (SPSS) version 25 (IBM, 2018).
